# 

**Published:** 1888-07

**Authors:** 


					﻿Vol. IX.
JULY, 1888.
No. 7.
TZETZE
inmiiiwracimoiifr
AY /VIOWT'MILY d(JuRNAL
DEVOTED TO
DENTAL AND ORAL SCIENCE.
W. XAVIER SUDDUTH, M. D . D. D. S
EDITOR-
The International Dental Journal Association,
PUBLISHERS,
51 West 37th Street, New York City.
$2.50 Per Annum in Advance, .... Single Copies, 25 Cents.
Entered at the Post-Office, Buffalo, N. Y., as Second-Class matter.
				

